# Role of condensates in modulating DNA repair pathways and its implication for chemoresistance

**DOI:** 10.1016/j.jbc.2023.104800

**Published:** 2023-05-09

**Authors:** Giuseppe Dall’Agnese, Alessandra Dall’Agnese, Salman F. Banani, Marta Codrich, Matilde Clarissa Malfatti, Giulia Antoniali, Gianluca Tell

**Affiliations:** 1 Laboratory of Molecular Biology and DNA repair, Department of Medicine, University of Udine, Udine, Italy; 2 Whitehead Institute for Biomedical Research, Cambridge, Massachusetts, USA; 3 Department of Pathology, Brigham and Women’s Hospital, Harvard Medical School, Boston, Massachusetts, USA

**Keywords:** condensates, DNA damage response, liquid-liquid phase separation, internal disordered regions, chemoresistance

## Abstract

For cells, it is important to repair DNA damage, such as double-strand and single-strand DNA breaks, because unrepaired DNA can compromise genetic integrity, potentially leading to cell death or cancer. Cells have multiple DNA damage repair pathways that have been the subject of detailed genetic, biochemical, and structural studies. Recently, the scientific community has started to gain evidence that the repair of DNA double-strand breaks may occur within biomolecular condensates and that condensates may also contribute to DNA damage through concentrating genotoxic agents used to treat various cancers. Here, we summarize key features of biomolecular condensates and note where they have been implicated in the repair of DNA double-strand breaks. We also describe evidence suggesting that condensates may be involved in the repair of other types of DNA damage, including single-strand DNA breaks, nucleotide modifications (*e.g.*, mismatch and oxidized bases), and bulky lesions, among others. Finally, we discuss old and new mysteries that could now be addressed considering the properties of condensates, including chemoresistance mechanisms.

Chemical changes in DNA can be highly harmful to living organisms. DNA, like other molecules, can undergo multiple chemical reactions. These reactions can occur spontaneously or as a result of exposure to chemicals or radiation (1). When these reactions result in altered DNA structure, including base excision and DNA breaks, DNA damage can block gene expression and replication negatively affecting cell homeostasis, function, and survival (2). Thus, DNA damage has to be promptly and properly repaired by the cells to allow for organism survival.

Decades of investigation into DNA damage repair pathways have shed light on diverse cellular pathways responsible to resolve various types of DNA damage. These pathways include base excision repair (BER), nucleotide excision repair (NER), homologous recombination (HR), mismatch repair (MMR), and non-homologous end joining (NHEJ) (3, 4). Dozens of different proteins must be efficiently recruited at DNA damage sites where these proteins work in coordination to allow for proper DNA damage repair. Structure-based studies and genetic studies have revealed interacting portions of proteins that allow for the recruitment of DNA damage repair proteins at sites of DNA lesions (5, 6, 7, 8, 9, 10). Moreover, great emphasis has been recently put also into disordered regions of proteins in concentrating molecules with shared function into subcellular compartments not enclosed within membranes, called biomolecular condensates (11, 12, 13).

In this review, we summarize key features of biomolecular condensates and DNA damage repair pathways with a focus on the involvement of biomolecular condensates in DNA damage repair. We also discuss open questions in the field of DNA damage repair that could now be addressed considering condensates and condensate properties.

## Biomolecular condensates and their assembly

A new emerging field of study in cell biology consists of the identification and characterization of biomolecular condensates. Condensates are defined as sub-cellular compartments that are not physically enclosed within membranes and can concentrate diverse molecules (including proteins and nucleic acids) (14, 15) to compartmentalize biochemical reactions (14, 16, 17, 18).

**Figure 1:**
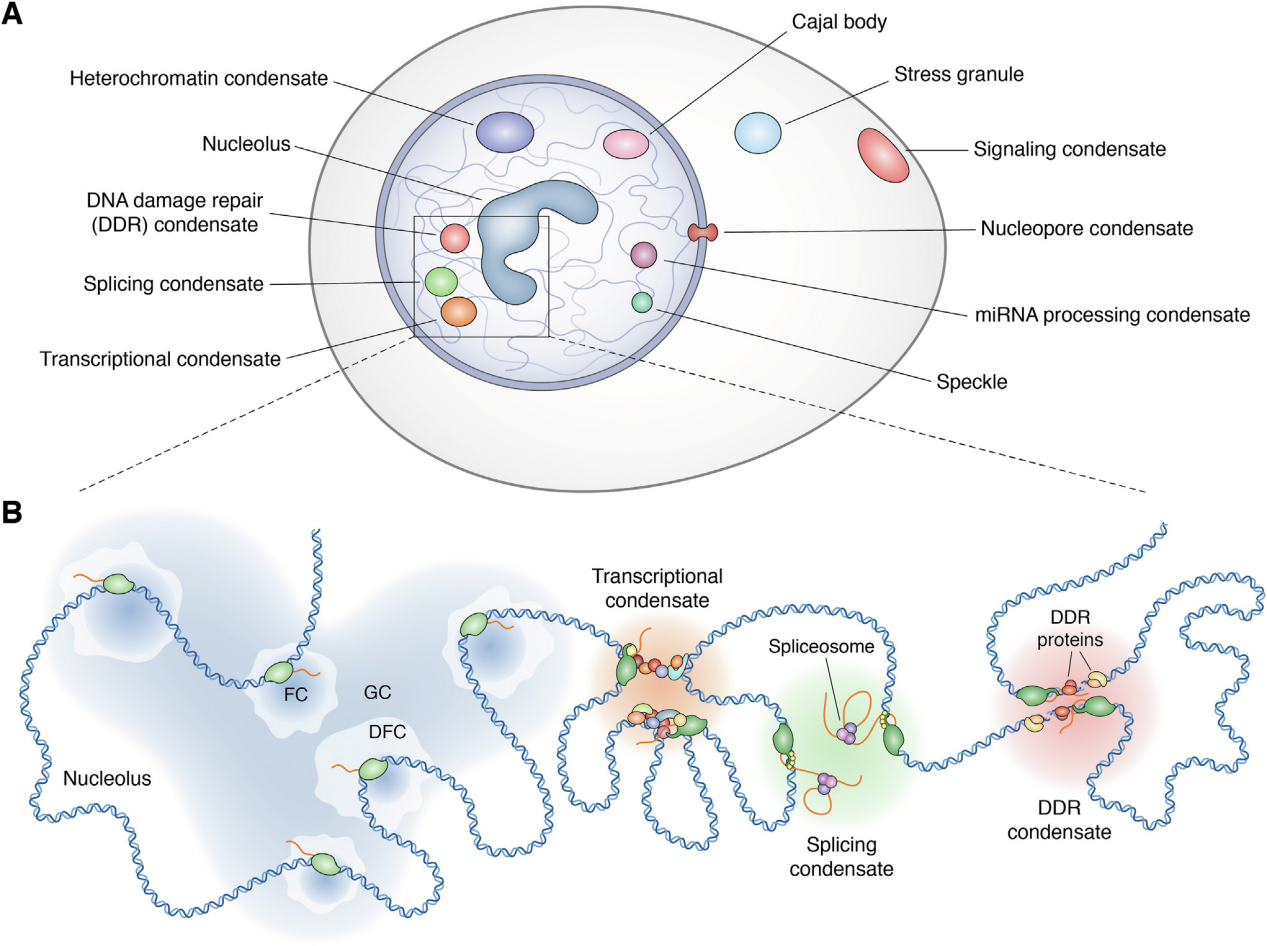
**Schematic overview of different biomolecular condensates identified in eukaryotic cells.***A*, Representation of different biomolecular condensates that can be found in the cytoplasm (Stress granules, signaling condensates), nucleus (Transcriptional condensates, Splicing condensates, DNA Damage Repair condensates, Nucleolus, Heterochromatin condensates, miRNA processing condensates, Cajal bodies) and in between (nucleopore condensates) of eukaryotic cells. *B*, Focus on transcription-associated condensates: Nucleolus (constituted by the FC where the active transcription of ribosomal DNA occurs through RNA Polymerase I; the DFC where the ribosomal RNA processing is orchestrated and the GC where take place the assembly of ribosomal subunits), Transcriptional condensates (where the initiation complex of RNA Polymerase II and several Transcription Factors and co-activators can be found), Splicing condensate (where elongating RNA polymerase II is recruited allowing for co-transcriptional splicing) and the DDR condensates (formed upon DNA damage thanks to the recruitment of DNA repair enzymes due to the transcriptional activity of RNA Polymerase II). DDR, DNA Damage Repair; DFC, Dense Fibrillar Component; FC, Fibrillar Component; GC, Granular Component.

The first condensates to be observed were the Nucleolus (19) and the Cajal bodies (20). Thanks to the advancement of microscopy technologies and a growing scientific interest in those compartments, many other condensates have been discovered, including transcriptional condensates (21, 22), nuclear speckles (23), splicing condensates (24), constitutive heterochromatin condensates (25, 26, 27), stress granules (28), signaling condensates (29, 30, 31, 32, 33, 34, 35, 36, 37, 38, 39), nuclear pore condensates (40), miRNA processing condensates (41), and DNA damage response (DDR) condensates (11, 42, 43, 44) (Fig. 1).

The physicochemical properties of condensates suggest that they may have diverse functions in cells (45). Condensates may allow for fast adaptive responses to changes in the environment. They can buffer concentrations of proteins; concentrate proteins to activate biochemical reactions; or sequester proteins to inactivate biochemical reactions. Thanks to their specific viscoelastic properties, condensates could generate mechanical forces. Condensates may also act as filters, for example, the nucleopore condensates, which permit or deny the entry of molecules into the nucleus (30, 45, 46, 47).

**Figure 2:**
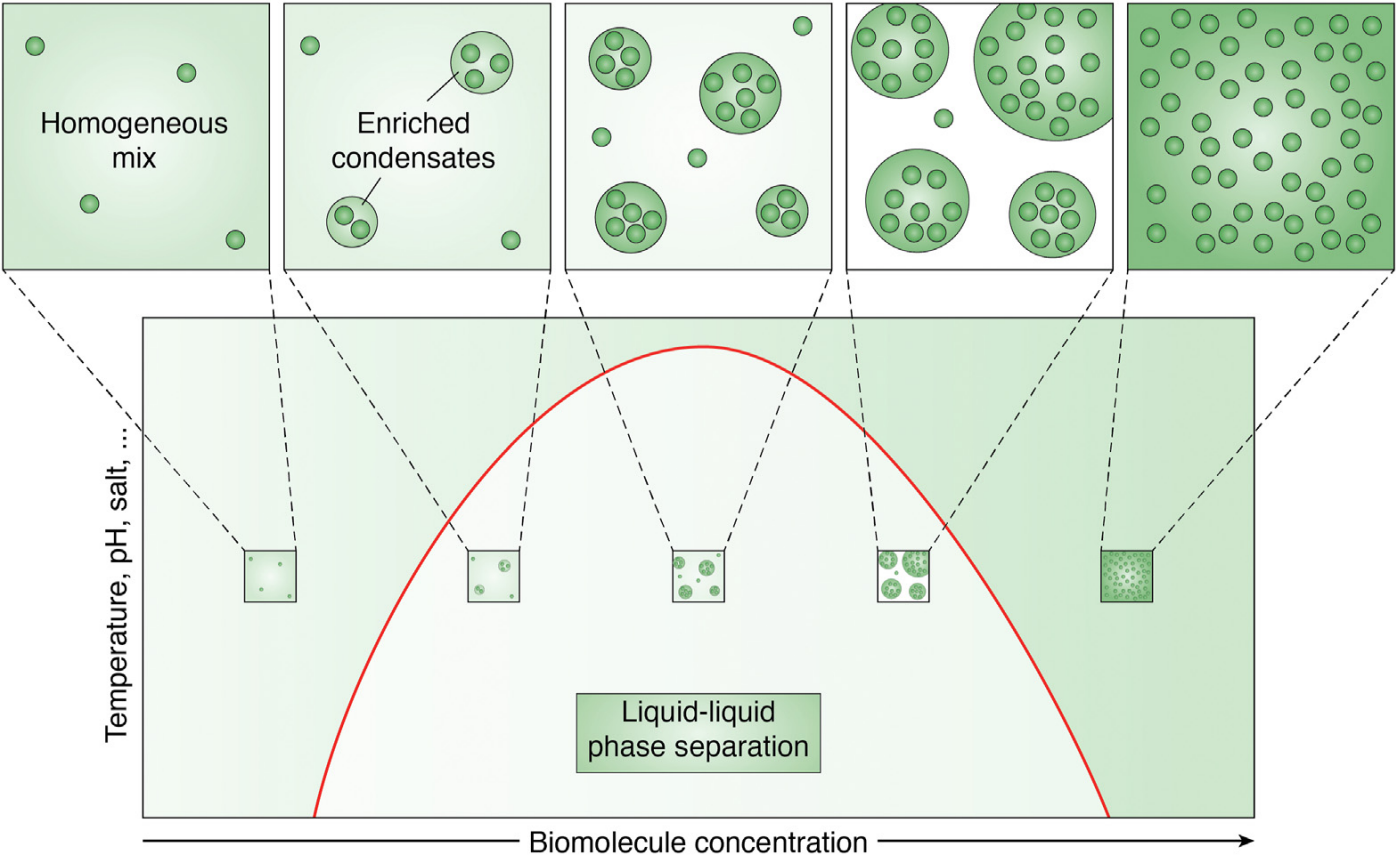
**Liquid-Liquid Phase Separation model.** Visual representation of the Liquid-Liquid Phase Separation model describable with a binodal curve (*redcurve*) that separates the 1 *versus* 2 phase regimes as a function of biomolecule concentration (x-axis) and system parameters such as temperature, pH, and salt’s concentration (y-axis). A lower concentration of biomolecules (*dark green*) will allow them to be homogeneously mixed in the solution (bottom *left panel*—widely diffuse *green color*) whereas higher concentrations will induce a phase separation leading to the formation of a more enriched (*green spheres*) and a less concentrated (*light green* or *white*) regions (*central panel*) until the condensed fraction will prevail (*bottom right panel*—*dark green* color with many biomolecules).

Among the different physical models that could explain how condensates are formed, one of them is Liquid-Liquid Phase Separation (LLPS) (48). LLPS can be described as a thermodynamically driven phenomenon consisting of de-mixing of a solution into two or more distinct liquid phases. In the 1940s, Flory and Huggins described the ability of polymers, such as proteins, to self-organize into discrete liquid-like droplets (49, 50), and over the last 10 years, many different proteins essential for life have been shown to be able to form dense phases resembling liquid-like droplets (14, 16, 21, 22, 24, 51, 52) dividing the cellular milieu into a “condensed phase” and a “dilute phase” (45) (Fig. 2).

Considering the potential impact of condensates in the biomedical field, it is important to determine what leads to the generation of those compartments. Over the years, different studies suggested that the formation of condensates is promoted by several factors, such as protein features (51, 53). Different protein domains are known to promote condensate formation, including structured domains and intrinsically disordered regions (IDRs)/low complexity domains (LCDs) (21, 53).

Among the different domains that can play a role in condensate behaviors, IDRs have been studied also in health and disease contexts (54). There are different types of IDRs, including elastin-like polypeptides (55) and prion-like domains (56), and can be enriched in charged amino acids (57, 58). Several studies tried to investigate the relevance of IDR composition to condensate formation. IDRs seem to preferentially form condensates with specific partners that show similar physicochemical characteristics and behavior, including charge–charge interactions and hydrophobic interactions (59). Considering the importance of protein–protein interactions for condensate formation, it is reasonable to think that it would be possible to artificially design molecules that will alter condensate formation for therapeutic purposes. Kameda and colleagues artificially designed small peptides that either promoted or impaired the formation of p53 condensates *in vitro*. This approach could also be taken to design peptide-therapeutics to alter condensate properties in cells (60).

IDRs are not the only drivers of condensate formation. Indeed, nucleic acids have been shown to play important roles in promoting the formation of several condensates, including nuclear speckles (61), stress granules (62), and transcriptional condensates (21). Notably, there is evidence of RNA involvement in controlling the formation and dissolution of condensates (63). RNA can interact with the charged residues of IDRs. One example is the RNA-mediated feedback model proposed by Young and colleagues (51) in which transcribed RNAs, such as short enhancer-RNA, initially stimulate the formation of transcriptional condensates by increasing the weak multivalent interactions between RNA and components of the transcriptional condensates. RNA appears to favor condensate formation until the overall charge of the condensate is zero (charge-balance model). At this point, further transcription of RNAs, including both enhancer-RNA and pre-mRNA, leads to the dissolution of the condensate. Moreover, also different structural protein subdomains can promote condensate formation, such as the SH3 domain (64) and the α-helical structure (65).

## Role of condensate formation in genome stability

**Figure 3:**
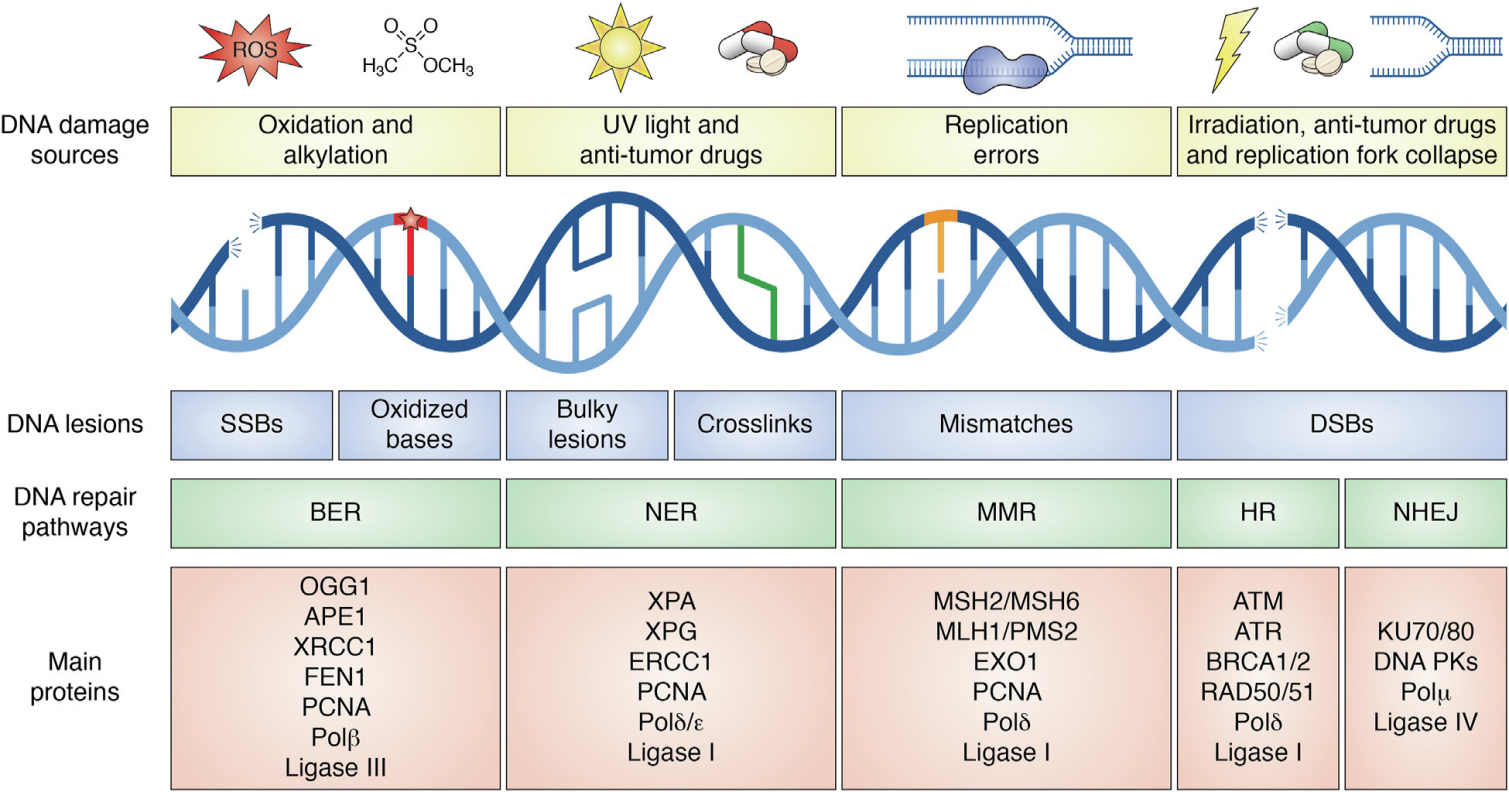
**Overview of DDR.** Schematic representation of DNA lesions caused by different damage sources and of respective DNA repair pathways. The major proteins involved in the DNA repair pathways are shown. BER, base excision repair; DSBs, double-strand breaks; HR, homologous recombination; MMR, mismatch repair; NER, nucleotide excision repair; NHEJ, non-homologous end joining; SSBs, single-strand breaks.

Genomic DNA is constantly subjected to endogenous and environmental threats, such as oxidative stress, alkylation, UV-damage, chemotherapeutics, and so on. These threats can damage the DNA, and unrepaired or incorrectly repaired DNA lesions could result in mutations or chromosomal rearrangements and aberrations. These alterations could lead to genomic instability, which is the basis of several human pathologies, ranging from cancer to neurodegenerative diseases (66, 67). Different DNA repair pathways are responsible for the specific recognition and repair (or tolerance) of specific DNA lesions. These pathways work together, as an orchestra, in playing the symphony of DDR in a cell-type-, cell-cycle stage- and chromatin context-dependent manner (68, 69, 70, 71, 72). There are three main classes of DNA repair pathways. The first class consists of DNA repair pathways that act at a single base level on non-distorting DNA lesions, such as the BER pathway. The BER pathway repairs the majority of oxidative and alkylating damages to the DNA through specific removal of the damaged base. It also repairs single-strand breaks (SSBs) through PARP recognition (73, 74, 75). The second class includes the NER pathway, which is involved in repairing distorting lesions such as those deriving from bulky adducts and UV-induced DNA damage. The NER pathway repairs DNA lesions by first excising and degrading a short stretch of ssDNA containing the damaged base and then by restoring the correct nucleotides in a polymerization-dependent manner (76, 77, 78). The third class of DNA repair pathways is the MMR, whose role is to correct DNA polymerase misincorporation errors ensuring the highest level of fidelity of the DNA replication process. MMR repairs several errors through excision and polymerization-dependent replacement of the DNA strand containing the mismatch (Fig. 3). These errors usually originate following: (i) mismatches occurring during DNA replication; (ii) a minority of single oxidative lesions to G, or (iii) the formation of bulges at highly repeated short oligonucleotide sequences, which are involved in trinucleotide expansion defects (79, 80, 81, 82). Finally, the most toxic DNA lesions, that is, the double-strand breaks (DSBs), are specifically repaired through cell cycle-dependent HR and cell-cycle–independent NHEJ, which are error-free and error-prone, respectively (83, 84, 85, 86, 87, 88, 89).

Mechanisms involved in the maintenance of genome stability have evolved over time. Although there is a certain specificity in the lesion recognition step, which is guaranteed by a plethora of different enzymes, one main feature of DDR is the crosstalk between the different DNA repair pathways. Indeed, during each step of the DNA damage repair process, intermediates are generated and they can become a substrate for enzymes that belong to different DNA repair pathways. One example is the crosstalk between the BER pathway with the NHEJ and HR pathways. When an abasic site is generated by a glycosylase, the BER enzyme APE1 generates a nick at the 5′ of the deoxy-ribose phosphate site thus producing an SSB. The replication of DNA containing SSB leads to a DSB. DSB can be further recognized and repaired by the NHEJ or the HR pathways. The crosstalk between the diverse DNA damage repair pathways ensures that they can compensate for each other and, more importantly, guarantees the maintenance of genome stability. An additional important aspect, which plays a pivotal regulatory role in coordinating the DDR kinetics, is represented by chromatin remodeling occurring at the site of the lesion, by the action of several chromatin remodelers, histone modifiers, and apical kinases such as ATM and ATR (72). Another layer of DNA repair regulation is exerted by chromatin organization, which is modulated by condensation mechanisms. Indeed, it has recently been suggested by many studies that the spatial organization of chromosomes into compartmentalized functional units, called topologically associating domains (TADs), depends on proteins such as cohesin and CTCF (90) and may depend on RNA produced by active transcription (91, 92) and proteins, which are assembled through nucleation mechanisms within condensates (93, 94). These condensation mechanisms, largely regulated by RNA molecules, allow reaching the local critical threshold concentration of specific enzymes into discrete chromatin compartments to favor the action of specific biological processes, including DDR, which can be potentially harmful to the cell if not properly spatially confined. Pessina *et al.* (95) showed that damage-induced long non-coding RNAs (dilncRNA) are actively synthesized at DSBs by RNA polymerase II and are necessary for DDR foci formation. The dilincRNAs are responsible for the molecular crowding of DDR proteins, such as 53BP1, into foci having LLPS condensates properties. Therefore, these condensation mechanisms may bring together signaling proteins and DNA repair enzymes to better control the kinetics of the DNA repair or the amplification of DNA damage signals (16).

**Figure 4:**
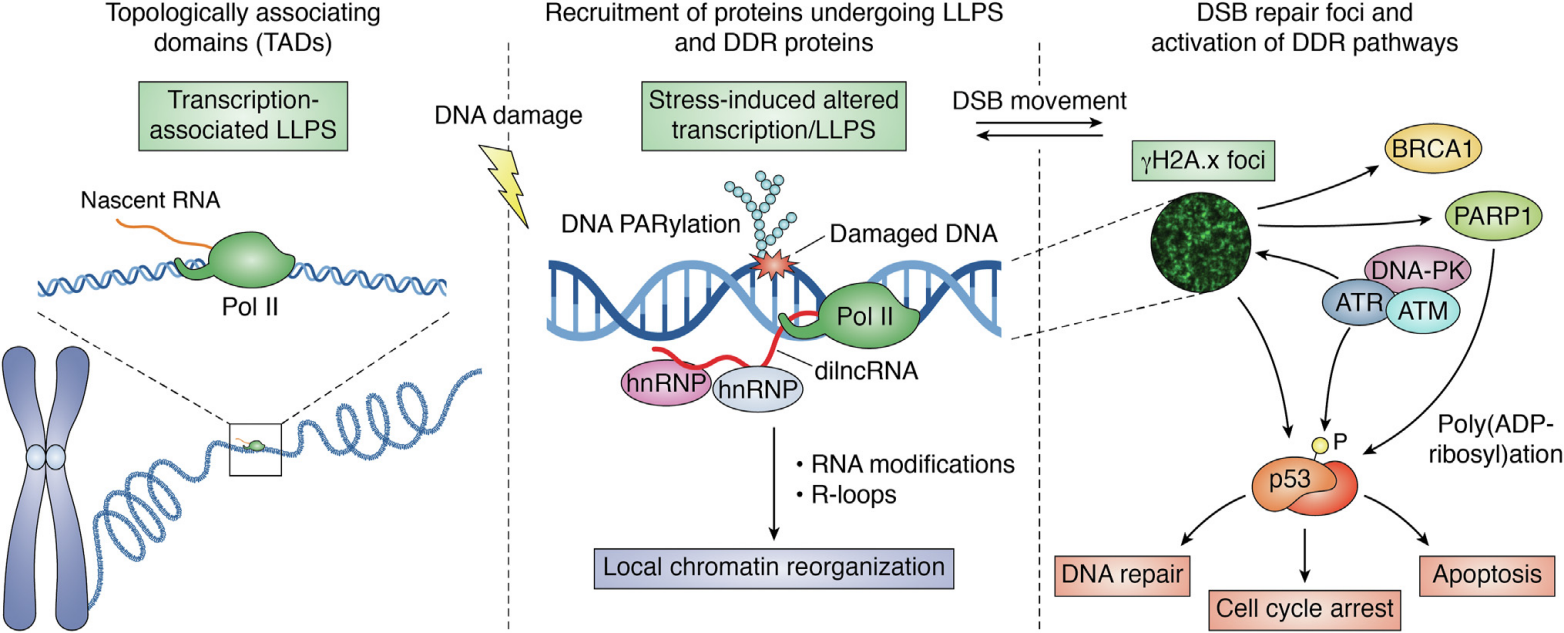
**Local chromatin organization and the role of RNA transcription and PARylation in DNA damage response.** The spatial organization of chromosomes into compartmentalized functional units called TADs, strictly depends on several proteins as well as RNAs produced by active transcription, which are assembled through nucleation mechanisms involving LLPS. Upon DNA damage, proteins undergoing LLPS and DDR proteins are recruited in stress-induced transcription-associated LLPS. DNA PARylated at the site of damage, as well as the nascent dilncRNA from Pol II, work for the recruitment of several ribonucleoproteins (hnRNPs), and for the induction of DNA repair. Moreover, a local chromatin organization is promoted through RNA modifications and R-loops generation. These processes encourage synchronously the signaling of the DNA repair and the activation of DDR pathways. Specifically, DNA-PK, ATM, and ATR protein kinases phosphorylate H2A.x stimulating the generation of γH2A.x foci, which opens chromatin for repair complex formation and signals the presence of DNA damage by activation of p53 and BRCA1. Both ATM and ATR are also responsible for the phosphorylation of p53 targeting it to the nucleus. While ATM is mainly involved in DSBs damage-induced repair, ATR is responsible for coordinating the repair of DSBs induced by replicative stress. Moreover, γH2A.x stimulates PARP1 which PARylates p53 protein. The effects of these orchestrated processes include DNA repair, cell cycle arrest, and lastly apoptosis. DDR, DNA Damage Repair; DSB, double-strand breaks; LLPS, liquid-liquid phase separation; TAD, topologically associating domains.

In the case of DSB repair, some recent works support the hypothesis that nuclear foci may promote the formation of condensates that could, in turn, favor the recruitment of DNA repair proteins at the site of the lesion. These involve many DNA repair proteins belonging to the HR pathway, such as BRCA1, RAD50, 53BP1, MRE11, RPA, and others, together with apical kinases, such as ATM/ATR, and the phosphorylated form of H2A.x histone (*i.e.*, γH2A.x). The MRE11/RAD50/NBS1 (MRN) complex is critical for the initiation of DNA damage response and DSB end resection. It has been recently shown (96) that the MRN complex interacting protein (MRNIP) forms liquid-like condensates to promote homologous recombination-mediated DSB repair through an intrinsically disordered region present in MRNIP protein. MRNIP condensates rapidly move to the damaged DNA to promote the binding of DSB by the MRN complex, thus triggering the autophosphorylation of ATM and the downstream DNA damage response signaling. Interestingly, clinical samples from patients with colon cancer and xenograft models confirmed a correlation existing between MRNIP expression and radioresistance, underlining a possible role of condensate formation in the onset of chemoresistance mechanisms. Phosphorylation of H2A.x histone and other histones is an early event in DSB repair, allowing recruitment of BRCA1 and 53BP1 to DSBs, giving rise to spreading waves of γH2A.x formation along several hundred/thousands of kilobases from DSB ends, with γH2A.x boundaries coinciding with TAD boundaries, involving CTCF and cohesin in controlling loop extrusion events (97, 98, 99, 100) (Fig. 4).

It is widely accepted that ATM and DNA-PK phosphorylate H2A.x in response to DSBs, while the H2A.x phosphorylation, induced by DSBs in nonreplicating cells, is ATR-independent (87, 101). ATR is involved in DDR activation promoted by DSB formation following replication stress and during meiosis in association with TopBP1 converging on many downstream effectors such as the checkpoint kinase Chk1 (102). It has been recently demonstrated that TopBP1 can extensively and reversibly self-assemble forming micrometer-sized condensates and undergo liquid-liquid phase separation *in vitro*. These condensates represent a molecular switch to amplify ATR activity to slow down replication forks by means of Chk1 phosphorylation (103). However, whether changes in chromatin organization could be affected by DNA repair through the formation of condensates is still poorly understood. Mono(ADP-ribosyl)ation (MARylation or MAR) and poly(ADP-ribosyl)ation (PAR-ylation or PAR) are post-translational modifications consisting of the addition of single or multiple ADP-ribose molecules to both proteins or DNA, catalyzed by (ADP-ribosyl)-transferase (ART) enzymes, including nuclear PARP-1 and -2 (104). PARP enzymes are known to catalyze the addition of MAR or PAR moieties at SSB and DSB foci, thus mediating the signaling of those damaging events (105). Interestingly, PARylation has been recently proposed as the main mechanism through which condensates could play a role in DDR, representing one of the main signals of genomic damage in cells (104, 106, 107, 108, 109, 110). This is not surprising since PAR is a nucleic acids-like negatively charged homopolymer and has properties suited for condensate formation. These modifications are also known to direct the formation of damaged DNA-enriched compartments and to recruit protein undergoing condensation, like FUS (109, 111, 112, 113), and the other FET family members (*i.e.* FUS, TAF15, and EWS) favoring the occurrence of molecular crowding phenomena at the damaged site (114). Recent studies demonstrated that PARP-mediated 53BP1 foci formation may occur through condensate-mediated mechanisms (42, 115), thus modulating the recruitment of p53 as a DNA damage-regulated transcription factor. Moreover, it was observed that active transcription of long non-coding RNA (dilncRNA) by RNA Pol II at the DSB damaged site is involved in 53BP1 partitioning into condensates (95). Similarly, TopBP1 has been recently demonstrated to form condensates and activate ATR kinase activity over Chk1 thus controlling the cell cycle entry (103). It has been recently described that human 53BP1 functions at heterochromatin regions undergoing LLPS with the heterochromatin protein HP1α, in a competitive manner, thus contributing to the maintenance of heterochromatin integrity. Interestingly, 53BP1 DSB-repair-deficient mutants, though proficient in LLPS, are able to rescue heterochromatin de-repression and protect cells from stress-induced DNA damage and senescence (116).

To date, although it is known that PARP interacts with several BER factors (*e.g.* XRCC1, POLβ and LIG3) modulating the activity of glycosylases (117, 118) and the 3′-exonuclease activity of APE1 (119) and that PARP inhibition significantly hampers the efficiency of the BER pathway (120, 121), there is no evidence for the formation of condensates important for the BER-mediated DDR. In a recent bioinformatics work (122), by analyzing the interactome of APE1 to look for clues of condensates formation in BER, we found that several APE1 interactors (such as FUS, SFPQ, NPM1, ESR1, APP, hnRNPA1, and LGALS3) are associated with cellular processes in which condensate formation and functionality have been proposed. This work might represent a paradigmatical pipeline for evaluating the relevance of condensates in DDR through the study of the DDR-proteins interactomes of specific enzymes having appropriate structural properties. It is noteworthy to mention that our previous hypothesis, regarding the possible concentration of APE1 into condensates, has been experimentally supported by a recent article showing that APE1 can form condensates *in vitro* and can concentrate in the nucleoli of cancer cells (123).

Condensate formation in DDR is required for the coordination of the kinetics of the DNA repair events. This was exemplified by the discovery that SLX4, a scaffolding protein coordinating the action of structure-specific endonucleases (SLX1, MUS81-EME1, and XPF-ERCC1) and other DNA repair proteins (*i.e.* TOPI) to ensure genome stability, is able to compartmentalize the SUMO system, thus promoting the resection of newly synthesized DNA as well as the processing of Topoisomerase 1 - DNA protein crosslinks (TOP1cc) (124).

It is interesting to note that telomeres could be also subjected to LLPS (125). The recombination-based alternative lengthening of telomeres (ALT) pathway is frequently used by Telomerase-free cancer cells to overcome limitations of replication due to the onset of an extrinsic senescent phenotype induced by oncogenic stimuli. The ALT-associated promyelocytic leukemia nuclear bodies behave as liquid condensates in response to telomere DNA damage. Zhang H. and colleagues recently suggested that APB coalescence drives telomere clustering independent of DNA repair factors, suggesting that these two processes may be regulated independently and providing a general framework to explain how chromatin condensates promote cellular functions (126). Another study showed that the APB-like condensates induce the formation of a small fraction of heterogeneous telomere lengths and of C circles, in the ALT-like phenotypes, in association with BLM. Moreover, these events are accompanied by ssDNA generation and RPA accumulation at telomeres, leading to mitotic DNA synthesis, which is mediated by RAD52 (127).

Notably, dynamic phase separation mechanisms are conserved also in prokaryotes. Recently, it has been demonstrated that single-stranded DNA-binding proteins (SSDBP), which are essential for the replication and maintenance of genome stability, form liquid-liquid phase-separated condensates in cellular-like conditions. *E. coli* cells use this mechanism to store a pool of SSDBP and SSDBP-interacting proteins and to enable rapid mobilization of this protein pool to keep genome stability by protecting exposed ssDNA (128).

## Analysis of IDR in DDR proteins

**Figure 5:**
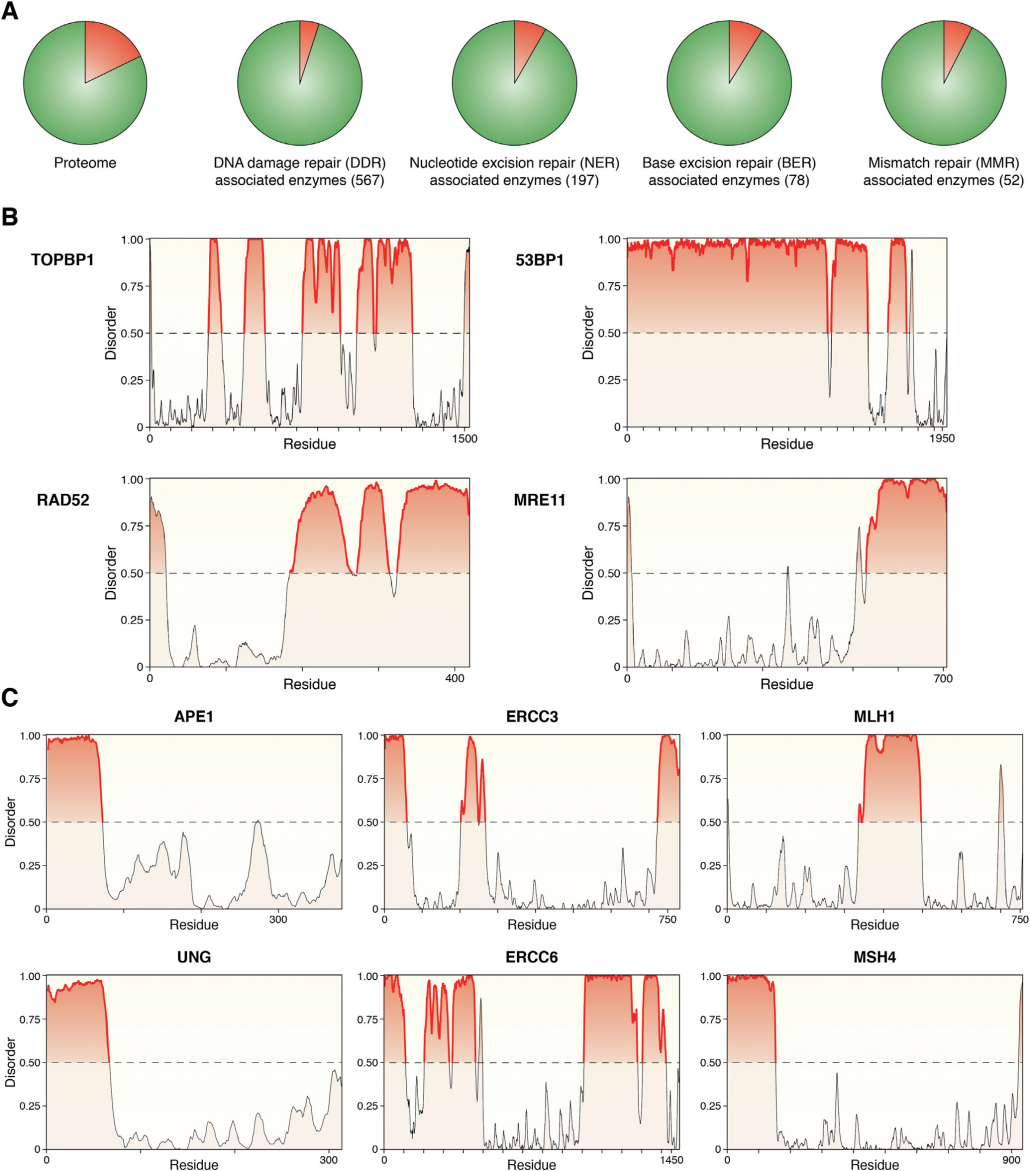
**IDR analysis on the main DNA damage repair enzymes.***A*, Pie-charts representing the percentage of DNA repair proteins with IDR longer than 50 amino acids. Within the whole proteome, 82% of proteins seems to have IDR longer than 50 amino acids (top left panel) whereas, out of 567 proteins belonging to different DDR pathways taken into consideration, 538 (94.5%) possess IDRs longer than 50 amino acids (*top right panel*). Among the 567 proteins investigated, 197 proteins belong to the Nucleotide Excision Repair (NER) pathway (*bottom left*), 78 of the Base Excision Repair (BER) pathway (*bottom center*) and 52 of the Mismatch Repair (MMR) pathway (*bottom right*), which showed to have 180, 71 and 48 proteins with IDR(s) longer than 50 amino acids respectively. IDRs were mapped using Metapredict (130), a consensus predictor that integrates several disorder predictors. IDRs were defined using a threshold of 0.2, which has been successfully used previously (165) and is within the recommended range of cutoffs suggested by the developers of the algorithm. *B*, Representative examples of IDR prediction of DSB enzymes TopBP1, 53BP1, RAD52, and MRE11. *C*, Representative examples of IDR prediction of APE1 and UNG, belonging to the Base Excision Repair pathway, ERCC3, and ERCC6, belonging to the Nucleotide Excision Repair pathway, MLH1 and MSH4, belonging to the Mismatch Repair pathway. All predicted structures in (*B*) and (*C*) were obtained using Metapredict (130, 131); y-axes represent the disorder content whereas the x-axes represent the aminoacidic residues of the proteins; each line in the x-axes marks 50 residues; highlighted in *red* the predicted IDR longer than 50 amino acids. DDR, DNA Damage Repair; DSB, double-strand breaks; IDR, Intrinsically Disordered Region(s).

The presence of IDRs, acquired during phylogenetic evolution in several DNA repair enzymes (129), largely thought to be responsible for the fine-tuning of their different activities through modulating protein–protein interaction with different partners, could now be explained as an evolutionary gain of function favored by their possible role in condensate formation. We thus investigated the hypothetical presence of IDRs in different proteins involved in DDR pathways, with a major focus on BER, NER, and MMR enzymes. For the first analysis, we took into consideration 567 proteins belonging to all the major DDR pathways and, using Metapredict (130), a consensus predictor that integrates several disorder predictors, we assessed that roughly 95% of the proteins taken in consideration possess IDRs longer than 50 amino acids. This outcome is interesting if we consider that the same analysis performed on the whole proteome shows that 82% of proteins contain an IDR longer than 50 amino acids. Intrigued by this result, we addressed the same question specifically for 78 BER-associated enzymes, 197 NER and 52 MMR enzymes (some of which are shared between those pathways). Not surprisingly, we found that 91% of BER, 91.4% of NER, and 92.3% of MMR enzymes possess IDRs longer than 50 amino acids (Fig. 5*A*), suggesting that most, if not all, enzymes involved in DDR might be active and might accomplish their enzymatic activity within the well-controlled and defined environment of condensates. Representative examples of IDR prediction on DDR enzymes using the same software (130, 131) are shown in Figure 5 (Fig. 5, *A*–*C*). In addition to this analysis, several pieces of evidence show that some of the above-considered proteins can bind RNA molecules (132, 133, 134) and can interact with other proteins already established to form condensates (114, 122, 135, 136, 137). Besides the predicted presence of IDRs within the aminoacidic sequence of those proteins, it will be necessary to assess whether these proteins can form condensates and to investigate the effective role played by those IDRs in the context of the enzymatic activity of those proteins within condensates.

## DDR enzymes-associated pathologies and possible aberration of condensate formation in human diseases

Alterations in the usual behavior of condensates have been suggested to be involved in the development of a variety of pathologies. The most studied pathologies thus far are neurodegenerative diseases and cancer (12, 138, 139, 140). Several studies have been performed to test whether condensate properties could be exploited to specifically target the aberrant condensates (15, 141, 142).

It is widely recognized that genome instability is one of the hallmarks of cancer (67). One of the plausible explanations for the increase in genome instability derives from the observation that, when some tumors start to develop, the activity of specific DDR pathways is often already compromised (143, 144).

Inhibitions of DDR condensate formation have been shown to coincide with impaired DNA damage repair. For example, PARP inhibitors, used to treat patients with cancer, prevent DNA repair condensate formation and impairs the DNA damage response (113). Inhibition of PARP can cause defects in both DDR and condensate formation. It would be quite relevant to develop tailored strategies to distinguish these two functions. Future studies that address this issue will have important implications for improved therapeutic approaches. Although at the moment there is not much evidence that mutations in different DNA damage repair genes may cause defects in condensate formation promoting genome instability and tumorigenesis, we suggest that this could be a plausible hypothesis to be tested in future works. Some examples that sustain this hypothesis include: (i) p53 R248Q mutation, which is highly associated with both breast and ovarian cancers and that decreases p53 DNA binding affinity, increasing the likelihood of p53 to form aggregates (145); (ii) the RGG motif mutation, occurring in FET-family associated proteins, that is associated with pathological protein aggregation (114); (iii) the mutation of TopBP1 in its conserved tryptophan, which is an essential residue for proper ATR activation in a condensate-dependent manner (103, 146).

Those considerations imply that cancers harboring mutations in DDR enzymes may rely more on the remaining functional DDR pathways to repair DNA lesions than cancers whose DDR enzymes are not mutated. Thus, they may be more susceptible to inhibitory compounds targeting the DDR pathways that remain functional (147). Therefore, the possibility that DDR enzymes concentrate in condensates participating into DNA repair within the condensates might be exploited to find different therapeutical approaches to specifically target and inhibit the DNA repair ability of cancer cells (141, 142, 147).

## Targeting condensate formation in DDR: a novel perspective to combat chemoresistance?

Based on the perspective that dysregulation of condensate formation has been associated with the pathogenesis and progression of several cancers (148), it is easy to deduce that the suppression of condensate formation and function by the means of selective disruption of their physicochemical properties have now deserved more attention as a new therapeutic target. Recent studies support the feasibility of targeting the unique biochemical environment of condensates in cancer cells (141, 149, 150). Different approaches have been proposed and employed to disrupt and/or modulate the formation and function of condensates implicated in disease (141, 151, 152). One way is to target key scaffold proteins that are essential for condensate formation or to inhibit the client–scaffold interactions such as FUS and other disordered proteins like EWS/FLI1, TAF15, and Sp1 that contribute to phase-separated transcriptional condensates at key oncogenes (141, 153). A second way would be to use molecules that target IDRs to prevent the partitioning of specific proteins into condensates. Although no clinically approved drug has been shown to act by specifically targeting cancer-promoting condensates thus far, studies showing the achievability of this option have been published (141, 154, 155). Drug targets included transcription factors (*i.e.*, MYC), hormone receptors, and nucleotide-binding proteins (*i.e.*, TDP-43). For example, the study from Girdhar and colleagues identified an acridine derivative, that is, AIM4, as an anti-TDP-43 aggregation molecule that binds to TDP-43 disordered region (154, 156). A third way is the use of drugs that target mediators influencing LLPS such as post-translational modifications of proteins or the epigenetic state of DNA and RNA, which are both essential for the nucleation of condensates (157, 158). RNA post-transcriptional modifications may impair the ability of RNA molecules to phase-separate. Additionally, epigenetic modifications via histone acetylation and methylation also contribute to tune phase separation of the chromatin. Considering DDR proteins, examples of these strategies have been described in the application of the therapeutic targeting of proteins involved in phase separation-driven DNA repair foci. As mentioned, DNA repair requires temporal and spatial coordination of diverse protein effectors which are recognized to be present in specialized condensates. The formation of these condensates is dynamically triggered by the deposition of PAR of protein enzymes and DNA to serve as a temporary scaffold to recruit low-complexity region-containing proteins in a time- and location-specific manner (42, 113, 137). Several groups have shown that PAR may seed the formation of compartments by dictating what client proteins are recruited (112). In this context, recent data have shown the therapeutic potential of PARP-1 depletion or PARP inhibitors, which have been clinically approved for cancer treatment and have been demonstrated to efficiently impair DNA repair condensate formation and hinder DDR (113). During DSB formation, after the earliest transient and reversible assembly of IDR proteins upon PAR, it has been demonstrated that FUS functions *in cis* to DNA damage, via a PARP-dependent mechanism to preserve genomic integrity (159). After genotoxic stress, the recruitment of FUS to PAR synthesized by PARP-1 at DNA damage sites generates dynamic compartments in which damaged DNA accumulates facilitating the recognition of damaged DNA by DNA repair factors. PARP-1 knockdown, the use of PARP-1 inhibitor (*e.g.* olaparib), or PARG inhibition have been shown to prevent the recruitment of DNA repair factors (113, 114). Furthermore, additional studies demonstrated that interfering with PAR-mediated condensates reduces the neurotoxicity of neurodegenerative diseases that are tightly associated with the dysregulation of condensates (160). In particular, since PAR can potentiate the formation of pathological aggregates containing amyloid proteins such as α-synuclein, FUS and TDP-43, the dysregulation of PAR with inhibitors (veliparib, rucaparib, and talazoparib) has been considered as a potential therapeutic strategy (161, 162). However, the molecular way in which PAR can modulate the dynamics and intracellular trafficking of client proteins is still unclear. It is conceivable that these inhibitors may affect the formation of condensates, nevertheless, future studies are needed to confirm or refute this hypothesis.

Another relevant research direction is to directly impair FUS function in LLPS. Recently, a screening for small molecules that selectively impair stress granule formation has identified lipoamide and lipoic acid as small molecules that target FUS reducing its propensity to aggregate thus opening new possible therapeutic routes (151, 163).

More intriguingly, the aberrant formation and behavior of condensates have been associated not only with various human pathologies but also functions in the development of chemoresistance (164). A study by Klein and colleagues has revealed that anti-neoplastic drugs, such as cisplatin and tamoxifen, become concentrated in specific condensates and that this occurs through physicochemical properties independent of the drug target. Notably, they showed that cisplatin partitions into transcriptional condensates containing Mediator Complex Subunit 1 (MED1) and this promotes drug activity. Interestingly, prolonged treatment of colon cancer cells with cisplatin led to the loss of MED1 from super-enhancer-associated transcriptional condensates causing their dissolution. Since various types of cancers rely on super-enhancer-mediated oncogene expression for their proliferation, the authors claimed that this association of cisplatin with transcriptional condensates and their subsequent dissolution could explain why DNA-modifying compounds, such as platinum drugs, are effective in a wide spectrum of anti-cancer therapies. Furthermore, the authors also showed that tamoxifen, which is used as an anti-estrogen compound to treat breast cancer, concentrates in MED1 condensates, leading to the eviction of estrogen receptor alpha from these compartments. Notably, they observed that MED1-overexpressing and tamoxifen-resistant breast cancer cells have larger MED1 condensates. As MED1 condensate size increases, the concentration of tamoxifen inside these condensates is decreased and, as a consequence, tamoxifen efficacy is decreased. This result can explain why tamoxifen resistance is often observed in association with MED1 overexpression. Thus, these two observations point out that small-molecule drugs can partition into cancer-associated condensates, and this partitioning has important implications for drug activity and drug resistance.

Although the development of condensate-related drugs is still in its early stage, the work of Klein and colleagues has important implications for future drug design. While deciphering the physicochemical features of condensates remains essential for the proper development of specific drugs, this needs to be integrated with chemical studies of compound properties that enable them to concentrate within specific compartments to improve therapeutic outcomes. These results suggest that selective partitioning and concentration of small molecules contribute to drug pharmacodynamics and that further insights into this phenomenon may facilitate advances in cancer therapy. At the moment, no clinically approved drugs are known to function by directly targeting disordered proteins; however, studies have shown that specific binding to disordered regions may be achievable. We refer interested readers to Ren *et al.* (150), Wang *et al.* (149), Mitrea *et al.* (141), and the references therein for a comprehensive review on this topic.

## Conclusive remarks and future perspectives

In this review, we provided several lines of evidence that support the potential involvement of condensate formation and condensate properties in the regulation of DDR. Further investigation is needed to detail if and how condensates, transcription, and RNA may impact DDR, possibly affecting profound chromatin compartmentalization and structural organization, and how these mechanisms could play a pathophysiological role in cells. These perspectives could help us understand missing aspects of cancer and neurodegenerative diseases, thus opening new opportunities for targeted therapies. Since anti-cancer therapies are partly based on inefficient DNA repair, further characterization of molecular dynamics associated with DNA repair mechanisms might uncover new oncological targets. Therefore, continuing to explore the possible condensate-related mechanisms driving early steps of DDR will certainly improve our knowledge of how to target deregulated processes responsible for genome instability, thus selectively impacting several human pathologies. Furthermore, the identification of modulators and strategies to regulate cancer-associated condensates represent important research directions in the future.

## Conflict of interest

The authors declare that they have no known competing financial interests or personal relationships that could have appeared to influence the work reported in this paper.
